# Magnitude of changes in patient symptom and medication scores in grass allergy immunotherapy trials: dependency on levels of pollen exposure

**DOI:** 10.1186/1710-1492-10-S2-A33

**Published:** 2014-12-18

**Authors:** Hendrik Nolte, Stephen R Durham, Harold S Nelson, David I Bernstein, Peter Creticos, Ziliang Li, Jens Andersen, Bente Riis

**Affiliations:** 1 Merck & Co., Inc., Whitehouse Station, NJ, USA; 2 Department of Allergy and Respiratory Medicine, Imperial College London, London, UK; 3 Departments of Medicine and Pediatrics, National Jewish Health, Denver, CO, USA; 4 Bernstein Allergy Group, Cincinnati, OH, USA; 5 Department of Medicine, Johns Hopkins University School of Medicine, Baltimore, MD, USA; 6 ALK-Abelló, Hørsholm, Denmark

## Background

Seasonal allergic rhinitis/rhinoconjunctivitis symptoms are dependent on pollen exposure and may impact the observed treatment effect of drugs used to treat seasonal allergic rhinitis. We conducted a post-hoc analysis to investigate the impact of pollen exposure on the overall magnitude of the recorded immunotherapy treatment effect across multiple seasons and trials of Timothy grass sublingual immunotherapy tablet MK-7243 (2800 BAU/75,000 SQ-T *Phleum pratense p* 5, Merck/ALK-Abelló).

## Methods

Data from seven North American and European randomized placebo-controlled trials of MK-7243 were included in the analysis (GT-02, GT-07, GT-08, GT-12, P05238, P05239, and P08067; data from GT-14 were not included since the observed lack of pollen-count/symptom relationship in this trial suggested etiology other than grass pollen exposure). Boundaries of three consecutive days with a pollen count ≥10 grains/m^3^ defined the grass pollen seasons (GPS). We assessed the correlation of between-treatment difference in total combined score (TCS; combined symptom+medication scores) per trial or trial year to the first-20-days-of-GPS cumulative grass pollen count and entire-GPS average pollen count.

## Results

Data from 1798 subjects on MK-7243 and 1765 subjects on placebo were included in the analysis. TCS for both groups increased with grass pollen counts. The treatment effect in TCS in each trial (or trial year) was correlated to the cumulative grass pollen count during the first 20 days of GPS (R^2^=0.803). A correlation was also seen between TCS and average pollen count over the entire GPS (R^2^=0.464).

## Conclusions

Post-hoc analysis of seven MK-7243 trials demonstrates that the magnitude of the treatment effect observed in the trials was highly correlated to the early-season grass pollen exposure observed in each trial. Therefore, differences in pollen exposure levels should be considered when comparing results among pollen immunotherapy trials and may contribute to observed differences in magnitude of efficacy between trials using the same immunotherapy formulation.

## Trial registration

ClinicalTrials.gov Identifiers: NCT00227279; NCT00408616; NCT00562159; NCT00550550; NCT01385371, 2 trials not registered

